# Endocrine and regenerative mechanisms of adipose-derived stem cells in female infertility

**DOI:** 10.3389/fendo.2025.1694025

**Published:** 2025-10-21

**Authors:** Zaher Merhi, Bhavika Garg, Jessica Haroun

**Affiliations:** ^1^ Department of Obstetrics and Gynecology, Division of Reproductive Endocrinology and Infertility, Albert Einstein College of Medicine, Bronx, NY, United States; ^2^ Department of Obstetrics and Gynecology, Division of Reproductive Endocrinology and Infertility, Maimonides Medical Center, Brooklyn, NY, United States; ^3^ Reproductive Endocrinology and Infertility, Rejuvenating Fertility Center, New York, NY, United States; ^4^ Medicine, University at Buffalo Jacobs School of Medicine and Biomedical Sciences, Buffalo, NY, United States

**Keywords:** adipose-derived stem cells, endometrium, ovary, embryo, infertility, regenerative medicine

## Abstract

Infertility remains a global health challenge, particularly in cases involving endometrial damage, diminished ovarian reserve, or poor embryo quality where conventional therapies often fail. Adipose-derived stem cells (ADSCs) have emerged as a promising regenerative option due to their accessibility, multipotency, and paracrine signaling capacity. This review evaluated preclinical and clinical studies investigating ADSCs and their derivatives for uterine, ovarian, and embryo applications in reproductive medicine. Literature searches were conducted in PubMed to July 2025, focusing on studies involving ADSCs, ADSC exosomes, and ADSC mitochondria in animal models and human studies. Results demonstrated that intrauterine ADSC administration improved endometrial thickness, vascularization, and receptivity, with some studies showing increased implantation and pregnancy rates in patients with thin endometrial lining thickness or Asherman’s syndrome. Ovarian applications showed partial restoration of function in premature ovarian insufficiency and chemotherapy-induced damage, with evidence of menstrual recovery, hormonal improvements, and enhanced folliculogenesis in both animal and early human studies. At the gamete and embryo level, ADSC-derived mitochondria, exosomes, and conditioned media improved oocyte maturation, reduced oxidative stress, enhanced blastocyst development, and increased embryo survival *in vitro*. Collectively, these findings highlight ADSCs’ therapeutic potential in addressing multiple infertility etiologies. However, current evidence is limited by small sample sizes, heterogeneous methodologies, short follow-up periods, and incomplete mechanistic insight. Most evidence to date comes from animal studies, while human clinical data remain limited to small early-phase trials. Large, well-designed clinical studies with standardized protocols and long-term safety evaluation are essential before ADSC-based therapies can be responsibly considered for full integration into assisted reproductive technologies.

## 1 Introduction

There is a growing prevalence of infertility among individuals of reproductive age with an expected continued increase through 2040 (1). Etiologies are multifactorial, but uterine abnormalities, diminished ovarian reserve (DOR) or premature ovarian insufficiency (POI), and impaired embryo quality are among the most difficult causes to manage (2). Conventional treatments—including hormone replacement, surgery, and assisted reproductive technologies (ART)—offer only partial solutions (3). For instance, endometrial damage in intrauterine adhesions or thin lining often resists medical and surgical interventions (4); ovarian insufficiency is typically managed with hormone replacement therapy (HRT) or oocyte donation (5), neither of which restores native ovarian function; and poor embryo quality remains a major contributor to recurrent implantation failure (RIF) despite optimization of *in vitro* fertilization (IVF) protocols. Thus, there is an urgent unmet need for innovative strategies that move beyond symptomatic management to true regenerative repair of reproductive tissues.

Recent advances in regenerative medicine have positioned mesenchymal stem cells (MSCs) as a promising therapeutic platform (6). Among the various MSC sources, adipose-derived stem cells (ADSCs) are particularly advantageous due to their abundance, minimally invasive harvesting, multipotent differentiation capacity, and robust paracrine activity (7–9). Beyond their ability to engraft and differentiate, ADSCs secrete a wide spectrum of bioactive molecules and extracellular vesicles (EVs, also called exosomes) and can donate functional mitochondria through intercellular transfer mechanisms (10–12). These unique properties suggest that ADSCs could address fundamental pathophysiological mechanisms underlying reproductive failure, including endometrial fibrosis, ovarian follicular depletion, and embryonic developmental arrest (10–12). Compared with bone marrow or umbilical cord–derived stem cells, ADSCs offer several distinct advantages: they are readily accessible through minimally invasive liposuction, yield higher numbers of viable cells, and display robust proliferative and paracrine potential. These features make ADSCs especially attractive for reproductive applications where repeated or autologous administration may be required (7–9).

Accumulating evidence from preclinical models and early-phase clinical studies supports ADSC-based interventions across multiple reproductive domains (13–15). In the uterus, ADSCs and their derivatives have been shown to increase endometrial thickness, promote angiogenesis, reduce fibrosis, and restore receptivity (16). In the ovary, intra-ovarian ADSC transplantation has demonstrated potential to partially restore hormone production and folliculogenesis in POI, while exosome- and mitochondria-based approaches may mitigate age- or chemotherapy-related decline in oocyte quality (17). At the embryo level, ADSC-conditioned media, lysates, and EVs have been reported to enhance oocyte maturation, improve blastocyst development, and increase cryotolerance *in vitro* (18).

Despite these promising advances, translation into routine clinical practice remains limited by small study sizes, heterogeneous methodologies, lack of standardized cell preparation protocols, and incomplete mechanistic understanding. The purpose of this review is to critically synthesize current preclinical and clinical evidence on the role of ADSCs in reproductive medicine, with a focus on their uterine, ovarian, and embryonic applications. By evaluating both the therapeutic outcomes and the limitations of existing studies, this review aims to clarify the translational potential of ADSCs as adjuncts to ART and to identify the key challenges that must be addressed before widespread clinical adoption.

## 2 Literature search and data extraction

A systematic literature search was conducted using PubMed database to identify relevant studies published up to July 2025. Search terms included combinations of “adipose-derived stem cells,” “endometrium,” “recurrent implantation failure,” “uterus,” “oogenesis,” “folliculogenesis,” “oocyte quality,” “granulosa cells,” “Asherman’s syndrome,” “oocyte,” “embryo,” “fertility,” “IVF,” and “implantation.”

Studies were included if they: (1) investigated ADSCs or their derivatives (exosomes, mitochondria, or conditioned media); (2) assessed reproductive outcomes in human or animal models; and (3) were original research articles, experimental studies, or clinical trials. Exclusion criteria encompassed review articles lacking new data, case reports with insufficient outcome measures, and studies utilizing non-adipose stem cell sources.

From each eligible study, key data were extracted, including study design, population or model, sample size, stem cell source, preparation and characterization methods, delivery route, dosage, timing of administration, endpoints, follow-up duration, principal findings, safety outcomes, and reported limitations. Both preclinical and clinical studies were examined, with outcomes categorized into uterine, ovarian, or embryo-related applications to enable structured synthesis and critical evaluation.

**Figure 1:**
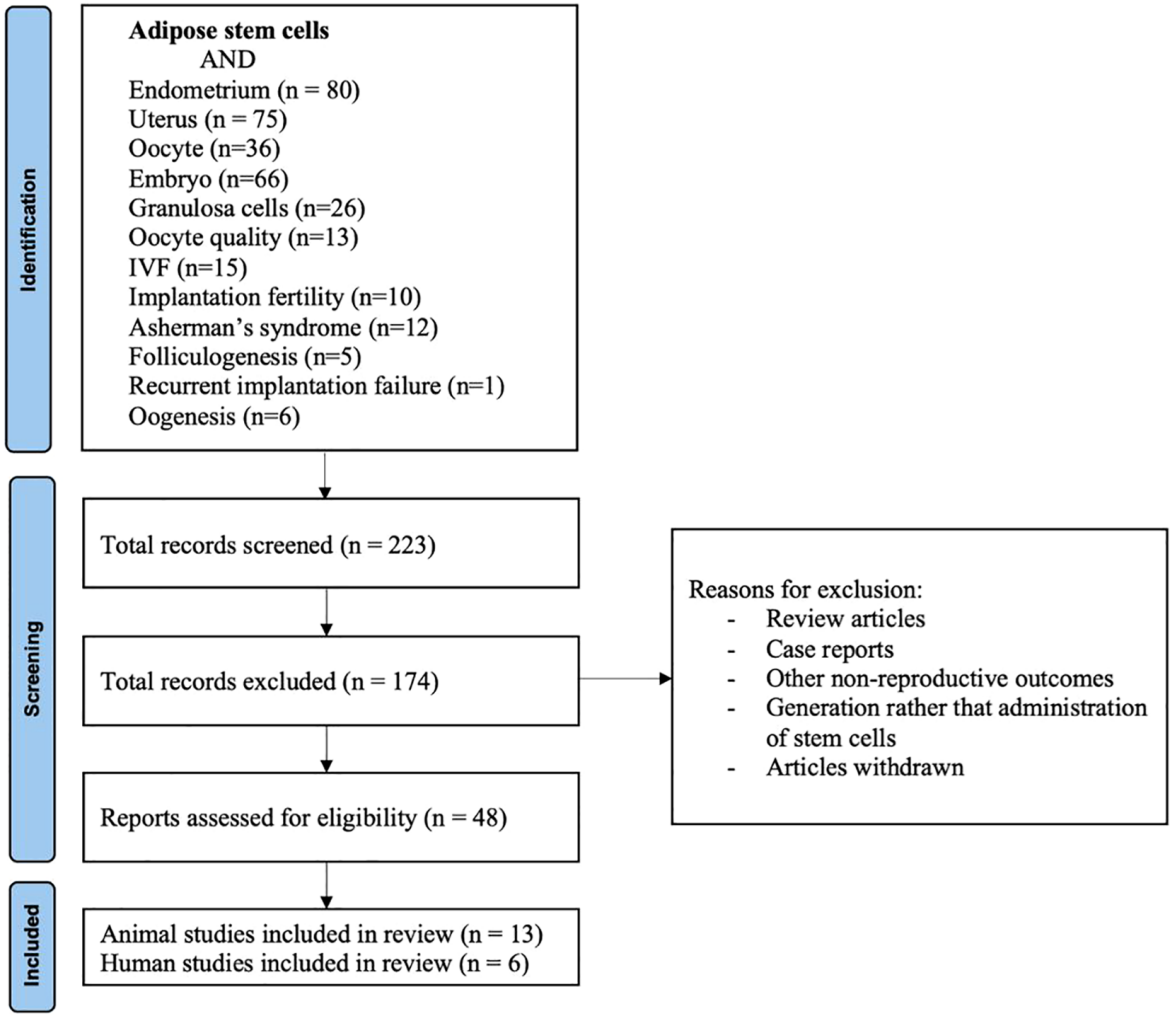
PRISMA flow diagram for study selection. The diagram illustrates the systematic literature search and screening process. Records (n= number of articles) were identified through PubMed. Titles and abstracts were screened for relevance, followed by full-text assessment of potentially eligible studies. Both preclinical (animal) and clinical (human) studies meeting the inclusion criteria were included in the qualitative synthesis.

The initially identified articles were screened by title and abstract. Full texts were reviewed when eligibility was unclear (**Figure 1**). Data extraction included study design, population/model, ADSC preparation, delivery route, dosage, outcomes, safety, and limitations.

## 3 Results and methodology

### 3.1 Uterine applications of ADCSs for injured endometrium

Intrauterine adhesions (IUA), often referred to as Asherman’s syndrome, occurs when the endometrial basal layer is damaged (commonly after dilatation and curettage, infection, or surgery), leading to fibrosis, abnormal uterine anatomy, menstrual disorders, infertility, and pregnancy loss (19). Thin endometrium remains a major challenge in reproductive medicine, as it is strongly associated with RIF and infertility. Several treatment protocols for inadequately thin endometrium included hormonal supplementation by estradiol (E2) (20), vasoactive agents such as aspirin (21), tocopherol (22), pentoxifylline (22), vaginal sildenafil (23), and others such as tamoxifen (24), granulocyte colony-stimulating factor (G-CSF) (25), and platelet-rich plasma (PRP) (19). Despite these measures, many patients do not respond to any or a combination of these treatments. Recently stem cell therapy has been proposed as a potential alternative for endometrial regeneration (16) (**Table 1**).

**Table 1:** Uterine applications of ADSCs for injured endometrium.

Study	Model/Population	Study design & intervention	Key results	Limitations
Han et al. (26)	Rat model of IUA	Ethanol-induced injury; 4 groups: sham, injury only, AHAM only, ADSC-seeded AHAM	↑ Endometrial thickness, ↑ gland counts, ↑ microvessel density, ↑ ERα, PR, integrin αVβ3, LIF; ADSCs localized in stroma	Xenogeneic model; no fertility outcomes assessed
Shao et al. (18)	Rat model of IUA	Chemical injury; ADSC transplantation *vs* PBS *vs* sham	↑ Endometrial thickness, ↑ glands, ↑ microvessels; ADSCs localized in basal layer; ↑ ERα/β, PR, integrin αVβ3, LIF; Fertility: 60% pregnancy *vs* 0% control; ↑ embryos implanted (3 *vs* 0)	Animal model only; 30-day follow-up; unclear optimal dosing; limited mechanistic insight
Dai et al. (27)	Rat endometrial injury	ADSCs seeded on porous collagen scaffold (CS/ADSC)	*In vitro*: promoted stromal proliferation, angiogenesis, ↓ fibrosis (↓ TGF-β1/Smad3), macrophage modulation. *In vivo*: ↑ EMT, glands, Ki67, microvessels, ↓ fibrosis; Fertility: 50% pregnancy, 28.4% implantation ratio; Transcriptomics: ECM & wound healing pathways enriched	Rat model; possible scaffold immune response; needs validation in large animals/humans
Yotsumoto et al. (28)	Mouse model of thin endometrium (ethanol-induced)	Fresh or frozen ADSCs transplanted intrauterine	↑ EMT, ↓ fibrosis, preserved gland/vascular architecture; Improved implantation: 47–69% *vs* 8% controls (77% in shams); Mechanism: ↑ VEGF expression; Frozen ADSCs effective; No added benefit with estrogen/hyaluronic acid	Mouse model; implantation rates below shams; limited mechanistic detail beyond VEGF
Hernández-Melchor et al. (29)	41 women with Asherman’s syndrome (refractory thin EMT <7 mm)	21 received intrauterine transmyometrial ADSCs (SVF); 20 controls	EMT ↑ from 4.9 ± 0.9 to 8.2 ± 1.9 mm; Implantation 66.7%; Clinical pregnancy 57.1%; Live birth 47.6%; Safe except minor bruising	Small sample; historical controls; confounding possible
Lee et al. (30)	6 women (20–44 yrs) with severe AS, thin EMT <5 mm	Autologous ADSC-SVF transplanted transcervically + estrogen	EMT ↑ from 3.0 ± 1.0 to 6.9 ± 2.9 mm; Improved trilaminar morphology; 2 resumed menses, 3 ↑ flow; 1 pregnancy (miscarriage at 9 wks); No adverse effects	Very small sample; no mechanistic analysis; 1 pregnancy outcome only

ADSC, Adipose-Derived Stem Cell; AHAM, Acellular Human Amniotic Membrane; AS, Asherman’s Syndrome; CS, Collagen Scaffold; ECM, Extracellular Matrix; EMT, Endometrial Thickness; ER, Estrogen Receptor; FSH, Follicle Stimulating Hormone; G-CSF, Granulocyte-Colony Stimulating Factor; IVF, In Vitro Fertilization; IUA, Intrauterine Adhesions; LIF, Leukemia Inhibitory Factor; PBS, Phosphate-Buffered Saline; PR, Progesterone Receptor; RIF, Recurrent Implantation Failure; SVF, Stromal Vascular Fraction; VEGF, Vascular Endothelial Growth Factor.

#### 3.1.1 Animal studies

In a rat model of IUA, a study (26) evaluated human ADSCs combined with acellular human amniotic membrane (AHAM) scaffolds for repairing injured endometrium. In that study, female Sprague–Dawley rats underwent ethanol-induced endometrial damage and received one of four interventions: sham surgery, injury without treatment, injury plus AHAM alone, or injury plus ADSC-seeded AHAM. The human ADSCs were isolated from human lipoaspirate, characterized for mesenchymal markers, and labeled with PKH26 for tracking. Fourteen days post-transplantation, the human ADSC-AHAM group demonstrated significantly greater endometrial lining thickness (EMT), higher gland counts, increased microvessel density, and elevated expression of receptivity markers: estradiol receptor (ER)-α, progesterone receptor (PR), integrin αVβ3, and leukemia inhibitory factor (LIF). Transplanted cells survived and localized to the endometrial stroma. The xenogeneic model and absence of fertility outcome assessment were clear limitations of the study even though it concluded that this scaffold-based delivery could enhance ADSC engraftment and repair in severe endometrial damage, potentially improving implantation rates in refractory cases.

Another study by Shao et al. (18) investigated ADSCs for repairing endometrial injury in a rat model of IUA. Female Sprague–Dawley rats with chemically induced damage received ADSC transplantation, PBS, or sham surgery. Over 30 days, ADSC-treated uteri showed greater thickness, more glands, and increased microvessel density versus controls. Transplanted ADSCs localized to the basal layer and expressed epithelial, stromal, and angiogenic markers, with higher estrogen receptor α/β, progesterone receptor, integrin αVβ3, and LIF expression. Fertility testing showed a 60% pregnancy rate in ADSC-treated rats versus 0% in PBS controls, with more embryos implanted (3 *vs*. 0). The authors concluded ADSCs aided regeneration through differentiation and paracrine effects (VEGF, cytokines). Limitations included use of an animal model, short follow-up (30 days), and incomplete mechanistic insight (18).

To enhance therapeutic retention, a study developed an ADSC-laden collagen scaffold (CS/ADSC) and tested it in a rat endometrial injury model (27). ADSCs were seeded onto porous collagen scaffolds before intrauterine implantation. *In vitro*, CS/ADSC promoted stromal proliferation, angiogenesis, reduced fibrosis via TGF-β1/Smad3 downregulation, and shifted macrophages toward an anti-inflammatory phenotype. *In vivo*, treated rats showed greater endometrial thickness, gland number, Ki67-positive cells, and microvessel density, with markedly less fibrosis. Fertility tests revealed a 50% pregnancy rate and 28.4% implantation ratio versus minimal implantation in controls. Transcriptomics confirmed effects on extracellular matrix and wound healing pathways. Collectively, CS/ADSC improved engraftment, reduced fibrosis, promoted angiogenesis, and restored fertility in a preclinical model. Limitations included reliance on a rat model, possible immune response to scaffolds, and need for validation in larger studies.

Building on these findings, Yotsumoto al (28). evaluated ADSCs in a mouse model of ethanol-induced thin endometrium. Fresh or cryopreserved ADSCs, containing stromal and vascular cell populations, were transplanted into the uterus and significantly increased endometrial thickness, reduced fibrosis, and preserved glandular and vascular architecture. Embryo transfer showed higher implantation rates in treated groups (47–69%) versus injured controls (8%), though still below sham levels (~77%). VEGF upregulation was identified as the primary mechanism, while other angiogenic mediators showed no change. Importantly, frozen ADSCs retained efficacy, supporting clinical practicality. These results suggest ADSCs offer a safe, minimally invasive, and cost-effective option for improving receptivity in refractory thin endometrium, though further clinical validation is needed.

These preclinical findings provided the foundation for subsequent translation into human studies, which are summarized below.

#### 3.1.2 Human studies

Extending the preclinical insights, a retrospective two-arm study (29) evaluated 41 women with Asherman’s syndrome after hysteroscopic adhesiolysis: 21 received intrauterine transmyometrial ADSC injections, while 20 served as untreated controls. ADSC therapy significantly increased endometrial thickness from 4.9 ± 0.9 mm to 8.2 ± 1.9 mm (p<0.0001). Implantation, clinical pregnancy, and live birth rates were markedly higher in the treated group (66.7%, 57.1%, and 47.6%) compared with baseline or controls. No major complications occurred beyond minor liposuction-related bruising. These findings suggest autologous ADSCs improve receptivity and IVF success in refractory Asherman’s patients, though the small cohort and reliance on historical controls limit interpretation, underscoring the need for prospective trials.

A pilot clinical study (30) investigated the use of autologous ADSC for endometrial regeneration in infertile women with severe Asherman’s syndrome. Six women aged 20–44 years with refractory thin EMT (<5 mm) after standard therapy were enrolled, and adipose tissue was harvested via liposuction, enzymatically processed, and transplanted transcervically into the uterine cavity using an embryo transfer catheter, followed by estrogen therapy. Five patients completed the study. The EMT significantly increased from a baseline of 3.0 ± 1.0 mm to 6.9 ± 2.9 mm (p = 0.043), with improved trilaminar morphology. Menstrual patterns also improved: two women with amenorrhea resumed scant menstruation and three with oligomenorrhea reported increased flow. Five patients underwent embryo transfer, resulting in one conception that ended in miscarriage at 9 weeks. No adverse events or immune reactions were observed. These findings suggested that autologous ADSC transplantation is feasible, safe, and may promote both structural and functional endometrial regeneration in severe Asherman’s syndrome, potentially improving receptivity. However, the study is limited by its small sample size, lack of specific stem cell characterization, absence of mechanistic analysis, and a single pregnancy outcome, underscoring the need for larger controlled trials to validate efficacy and establish standardized protocols.

### 3.2 Ovarian applications of ADSCS

Premature ovarian insufficiency, defined as the partial loss of ovarian activity before age 40 and affecting up to 3.5% of reproductive-age women, remains a major cause of infertility with limited treatment options beyond HRT and oocyte donation (31). At present, POI remains irreversible, and while long-term HRT can alleviate menopausal symptoms, it does not effectively prevent or delay premature ovarian aging in women. This underscores a need for more effective treatment strategies (32).

#### 3.2.1 Intraovarian administration of ADSCs for premature ovarian insufficiency

In a first-in-human, non-randomized, phase I clinical trial (33), nine women with idiopathic POI were enrolled and divided into three groups (n=3 in each group) to receive intra-ovarian transplantation of autologous ADSCs at escalating doses of 5×10^6^, 10×10^6^, or 15×10^6^ cells. ADSCs were isolated from abdominal adipose tissue via liposuction, processed under GMP conditions, characterized by flow cytometry, and transplanted unilaterally under transvaginal ultrasound or laparoscopy guidance. Participants were followed at intervals up to 12 months, with safety as the primary outcome, and secondary measures including resumption of menstruation, hormonal changes (follicle-stimulating hormone [FSH] and anti-Mullerian hormone [AMH]), ovarian volume, and antral follicle count. No immediate or delayed adverse effects were observed, confirming procedural safety. Four patients resumed menstruation (two within one month and two within two months), sustained for several cycles, and four showed serum FSH declines below 25 IU/L, some persisting for up to a year. Ovarian volume, AMH, and follicle counts remained variable without significant group differences. Overall, ADSC transplantation appeared safe, feasible, and associated with menstrual recovery and partial hormonal improvement. However, limitations include the very small sample size, absence of a control group, non-randomized design, heterogeneous responses, and lack of mechanistic insight into follicular renewal. Larger, controlled studies are clearly needed to validate these preliminary findings and optimize dosing strategies.

Chemotherapy-induced ovarian failure represents a major cause of infertility, and current options for restoring ovarian function are limited (34). Building on clinical observations in POI, chemotherapy-induced ovarian failure has also been studied as a target for ADSC therapy. In a CTX-induced damage model in rats and mice (15), intraovarian ADSC transplantation significantly increased endothelial cells, restored follicle and corpus luteum counts, and improved litter size compared with saline controls (13.6 *vs*. 9.4 pups). No malformations or tumorigenesis were observed in F1 or F2 offspring. ADSCs secreted higher VEGF, IGF-1, and HGF than fibroblasts, with transplanted ovaries showing upregulation of these cytokines and StAR mRNA, consistent with angiogenesis and follicular support. FISH confirmed ADSCs localized to the thecal layer but not follicles, indicating paracrine rather than germline effects. Administration of VEGF, IGF-1, or HGF alone provided only partial recovery, underscoring synergistic action. Overall, ADSCs restored ovarian function safely through angiogenic and trophic mechanisms, though findings remain limited to rodent models.

In summary, preclinical work has primarily evaluated ADSCs in POI and chemotherapy-induced ovarian failure. In rodent models, intraovarian ADSC transplantation restored follicular numbers, increased angiogenesis, and improved litter sizes, largely through paracrine release of VEGF, IGF-1, and HGF. These trophic effects supported granulosa cell survival and follicular development while avoiding tumorigenesis across generations. Importantly, transplanted ADSCs localized to stromal and vascular compartments, suggesting that benefits derive from microenvironmental modulation rather than direct germline contribution. Systemic delivery routes, although less studied, also warrant investigation given their potential translational convenience.

#### 3.2.2 ADSC’s mitochondrial replacement therapy for poor oocyte quality

**Figure 2:**
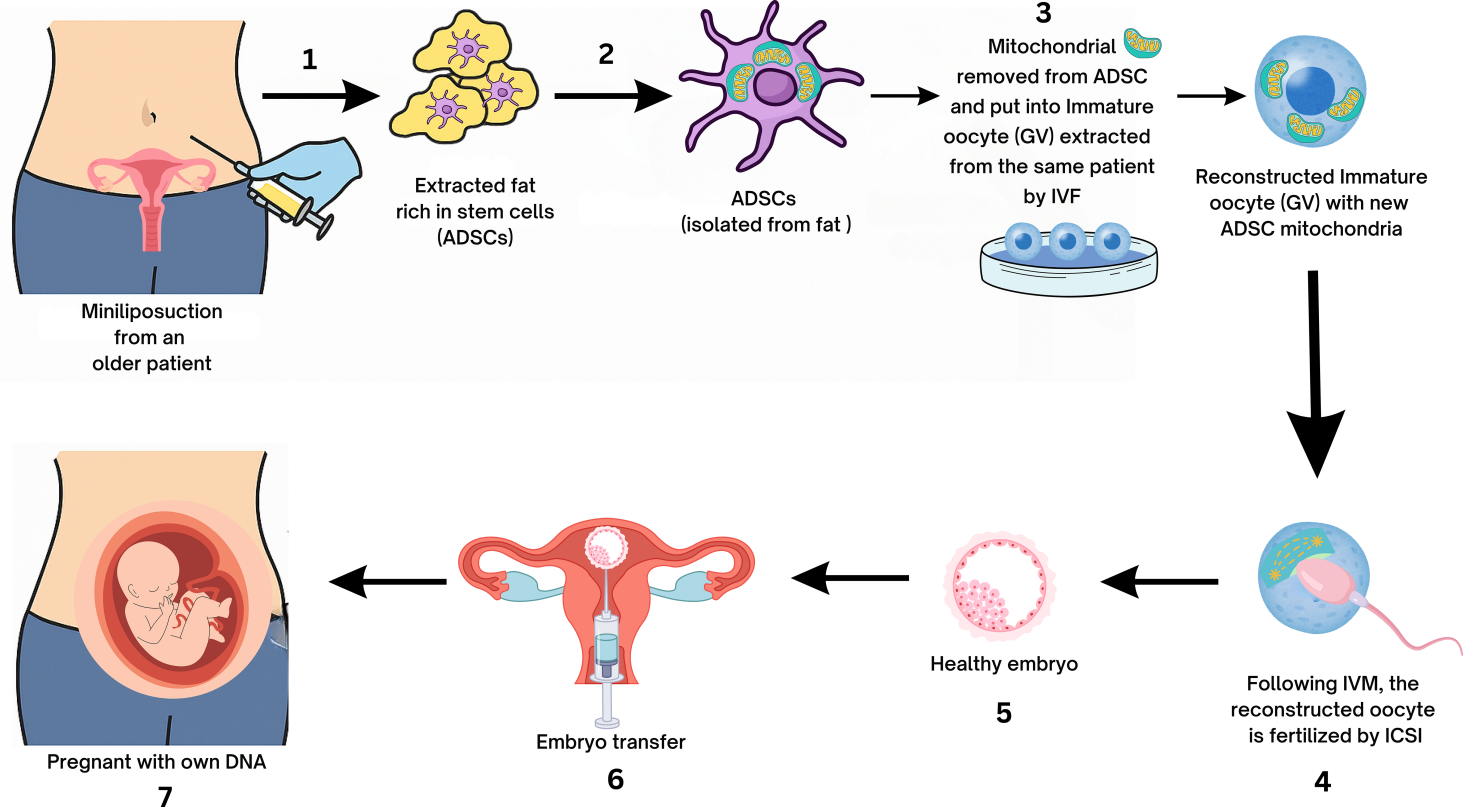
Autologous adipose-derived stem cells (ADSCs) mitochondrial replacement therapy (MRT) for age-related oocyte quality decline. Diagram illustrating the comprehensive experimental workflow for autologous MRT from ADSC into GV oocytes - detailing the sequential steps from adipose tissue procurement, ADSC isolation, and subsequent mitochondrial extraction, to the precise microinjection of isolated mitochondria into GV oocytes. GV, germinal vesicle; IVF, in vitro fertilization.

Maternal aging is a leading cause of infertility, primarily due to declining oocyte quality associated with mitochondrial dysfunction, reduced ATP production, spindle defects, and chromosomal missegregation (35). While heterologous MRT have raised concerns about heteroplasmy and genetic incompatibility (36), autologous mitochondria from ADSCs offer a potentially safer alternative. In one experimental study (17), ADSCs from aged mice were cultured, and their mitochondria extracted and microinjected into germinal vesicle (GV) and metaphase II (MII) oocytes. Mitochondrial morphology, mtDNA copy number, and spindle alignment were evaluated, and fertility was tested using ICSI and embryo transfer. ADSC mitochondria retained normal ultrastructure and supplementation significantly increased mtDNA content [(12.47 ± 4.16) ×10^4^ *vs*. (8.38 ± 1.99) ×10^4^], improved spindle integrity, reduced aneuploidy (4/12 *vs*. 11/18), and enhanced blastocyst formation (30% *vs*. 15%). Embryo transfer produced eight live pups from 51 embryos in the supplemented group compared with one from 50 controls. These results demonstrate that autologous ADSC-derived mitochondria can rescue oocyte quality and restore fertility in aged mice, supporting translational potential (**Figure 2**). Limitations include reliance on an animal model, lack of long-term offspring safety data, and uncertainties regarding scalability, ethics, and clinical application in older women.

Another study (12) screened autologous donor cells—granulosa cells (GC), ADSC, bone marrow-derived mesenchymal stromal cells (BMSC), and urine-derived stem cells (USC)—to evaluate mitochondrial suitability for oocyte cytoplasmic transfer. Using electron microscopy, mtDNA quantification, membrane potential, reactive oxygen species (ROS) levels, and metabolic assays, USC mitochondria were found to most closely resemble oocytes, with high content, robust activity, low oxidative stress, and relative resistance to aging effects. Whole mitochondrial genome sequencing confirmed biosafety with no pathogenic variants. To assess functional effects, USC mitochondria were co-injected with sperm during ICSI into discarded immature oocytes matured *in vitro* from both young (<35) and advanced-age (≥35) IVF patients. Compared to conventional ICSI, the USC MRT significantly increased embryonic mitochondrial content, improved membrane potential, reduced ROS and cytosolic Ca²^+^ levels, and enhanced developmental outcomes, especially in aged oocytes. Morphological improvements included higher rates of 7–10 cell embryos and good-quality day-3 embryos, as well as a trend toward improved blastocyst formation. Importantly, euploidy rates were higher in aged embryos after mitochondrial transfer (77.8% detection, with 28.6% euploid embryos *vs.* none in controls). These findings suggest that non-invasively derived USC mitochondria are an optimal autologous donor source, capable of rescuing mitochondrial dysfunction in oocytes and improving embryo quality without third-party genetic risks. However, limitations include reliance on discarded immature human oocytes, small sample sizes, lack of embryo transfer and clinical outcome data, and the need for long-term safety validation before translation to clinical ART practice.

In summary, clinical evaluation remains limited but encouraging. A first-in-human, phase I trial of intraovarian autologous ADSC transplantation in idiopathic POI reported safety, partial hormonal recovery, and resumption of menses in a subset of patients. While these results highlight feasibility, the trial was small, non-randomized, and heterogeneous in response. Further early-phase studies are needed to optimize dosing, delivery routes, and patient selection. To highlight the current state of clinical translation.

#### 3.2.3 ADSC’s exosomes for poor oocyte quality

The EVs, some of which are called exosomes, are secreted by stem cells and play critical roles in cell-to-cell communication, carrying proteins, nucleic acids, and metabolites that regulate development and survival (37, 38). The embryotrophic potential of ADSCs has been suggested in multiple species (39). A study (39) examined whether ADSC-derived EVs enhance porcine embryonic development *in vitro*. ADSCs were isolated, confirmed by flow cytometry and differentiation assays, and cultured to harvest EVs, which were characterized by nanoparticle tracking, cryo-TEM, and Western blot. Parthenogenetic embryos were cultured in EV-supplemented or control media, and outcomes assessed included development, oxidative stress, mitochondrial activity, apoptosis, gene expression, and proteomics. EV supplementation significantly improved blastocyst formation (28.6% *vs*. 24.8%), increased glutathione, reduced ROS, and enhanced mitochondrial activity. TUNEL assays showed fewer apoptotic cells, with blastocysts containing more cells. qPCR revealed upregulation of pluripotency genes (OCT4, NANOG, SALL4) and downregulation of BAX. Proteomics identified 1,543 proteins, with enrichment in cell cycle pathways; SRC abundance suggested an SRC-AKT-CDK1 axis promoting cell division. Overall, EVs improved blastocyst quality by reducing oxidative stress and apoptosis. Limitations include reliance on porcine parthenogenetic embryos, absence of *in vivo* validation, and uncertain clinical applicability.

Exosomes from ADSCs are key mediators of paracrine effects and can regulate autophagy via the AMPK/mTOR pathway, critical for granulosa cell (GC) survival and folliculogenesis (13). In a cyclophosphamide-induced POI mouse model, treatment with ADSCs, ADSC-exosomes, or controls was compared (13). Exosomes were characterized by TEM, nanoparticle analysis, and surface markers (CD63, CD81, TSG101, HSP70). Outcomes included ovarian morphology, hormone levels, oxidative stress, follicle counts, GC apoptosis, autophagy markers, and AMPK/mTOR signaling. Exosome treatment restored ovarian weight, follicle number, and histology; increased E2; decreased FSH; reduced ROS and MDA; and elevated SOD. TUNEL assays confirmed reduced GC apoptosis, while Western blotting showed downregulation of Beclin-1 and LC3II/LC3I with upregulation of Bcl-2, indicating autophagy suppression. Exosomes also decreased p-AMPK and increased p-mTOR, reversing POI-associated activation. *In vitro*, exosomes improved viability and reduced apoptosis in CTX-injured KGN cells, effects abolished by rapamycin. Overall, exosomes protected against chemotherapy-induced POI by reducing oxidative stress, apoptosis, and AMPK/mTOR-driven autophagy. Limitations include reliance on a murine xenograft model, short follow-up, and lack of reproductive outcome data.

**Figure 3:**
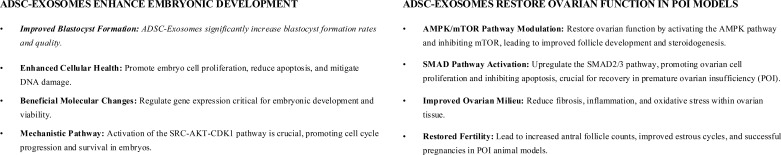
Adipose-derived stem cell (ADSC) exosomes. Exosomes are cell-secreted nanoparticles that mediate critical intercellular communication by transporting bioactive molecules including proteins, nucleic acids, and metabolites. ADSCs provide an abundant, accessible source of exosomes with demonstrated embryotrophic and ovarian restorative potential. POI, premature ovarian insufficiency.

Building on prior work, another study (40) examined whether human ADSC-derived exosomes could restore ovarian function in chemotherapy-induced POI via the SMAD pathway. Exosomes were isolated from ADSC-conditioned medium, characterized, and injected into ovaries of POI mice, with parallel *in vitro* experiments using GCs from POI patients. Outcomes included follicle counts, hormone levels, GC proliferation/apoptosis, marker expression, and SMAD2/3/5 signaling. Exosome treatment restored follicle numbers across all stages, normalized E2, AMH, and FSH, and improved ovarian histology. *In vitro*, treated GCs showed enhanced proliferation (76% *vs*. 8% in controls), reduced apoptosis (4% *vs*. 74%), and normalized expression of FSHR/AMH and FOXL2/CYP19A1. Mechanistically, exosomes upregulated SMAD2/3/5, while SMAD knockdown abolished protective effects and increased apoptosis genes. These results indicate that ADSC exosomes improve ovarian function by promoting folliculogenesis, normalizing hormone secretion, and protecting GCs through SMAD activation (**Figure 3**). Limitations include reliance on a murine POI model, short-term follow-up, and lack of detailed exosomal cargo characterization.

### 3.3 Embryo applications of ADSCS

Optimizing *in vitro* maturation (IVM) is critical for ART, yet conventional media often lack paracrine factors present *in vivo*. Human ADSCs, which secrete cytokines and growth factors, may provide this support through conditioned medium (ADSC-CM). A study (41) tested porcine cumulus–oocyte complexes in control media, ADSC co-culture, or ADSC-CM supplementation. Outcomes included maturation, embryo development, and gene expression. Both ADSC conditions significantly increased nuclear maturation (~85% *vs*. 78% control), cumulus expansion, and cleavage rates, with a trend toward higher blastocyst formation (27% *vs*. 18%). Blastocysts also contained more cells (~66–68 *vs*. 46). ELISA confirmed enriched VEGF, bFGF, IGF-1, IL-10, and EGF in ADSC-CM, with reduced ROS. Gene and protein analyses showed upregulation of receptors, anti-apoptotic BCL2, cumulus expansion genes, and oocyte markers GDF9 and BMP15, strongest with ADSC-CM. Overall, ADSC-CM improved oocyte competence and embryo quality, suggesting utility as an IVM supplement. Limitations include use of porcine oocytes, parthenogenetic embryos, and absence of *in vivo* validation.

Improving efficiency and cryotolerance of bovine *in vitro*–produced embryos remains challenging. Serum enhances blastocyst formation but increases lipid accumulation and reduces survival after freezing. ADSCs, which secrete regenerative factors, were tested as lysate supplements. In one study (42), bovine ADSCs were isolated, confirmed by immunophenotyping, and disrupted by freeze–thaw to prepare lysates. Fibroblast lysates and fetal calf serum (FCS) served as controls. Bovine IVF embryos were cultured in synthetic oviductal fluid with these supplements, and outcomes included cleavage, blastocyst rates, cell counts, cryotolerance, lipid accumulation, and gene expression. ADSC lysate (10%) significantly increased blastocyst formation (43.0% *vs*. 29.9%, p<0.05), with effects paralleling 10% FCS. Unlike FCS, ADSC lysate maintained lower lipid levels, improved viability, and conferred superior cryotolerance with higher survival and hatching after thawing. Gene expression was unchanged, indicating preserved developmental programming. Overall, ADSC lysate improved embryo development and post-thaw viability without the drawbacks of serum, though validation *in vivo* and mechanistic clarification are still needed.

Despite advances in defined embryo culture media, co-culture with somatic cells remains common in bovine embryo production to mimic the reproductive tract. Traditionally, GCs are used, but mesenchymal stem cells may offer superior paracrine support. A study (43) compared bovine ADSC co-culture with granulosa cells or conditioned medium. In Experiment 1, zygotes were cultured in SOF alone, SOF preconditioned with ADSCs, or directly with ADSCs (10³ or 10^4^ cells/mL). In Experiment 2, embryos were co-cultured with ADSCs (10^4^ cells/mL) or GCs, and outcomes included cleavage, blastocyst rates, cell counts, and gene expression. Co-culture with 10^4^ ADSCs/mL significantly increased blastocyst rates (45.9%) versus SOF (19.0%) or conditioned medium (25.0%), with higher cell numbers and upregulation of pluripotency (POU5F1) and metabolic (G6PDH) genes. Unlike GC co-culture, ADSCs did not increase HSP70, suggesting absence of added stress. The authors concluded ADSCs enhanced embryo quantity and quality through paracrine modulation. Limitations include reliance on a bovine model, lack of *in vivo* validation, and incomplete identification of secreted factors.

**Figure 4:**
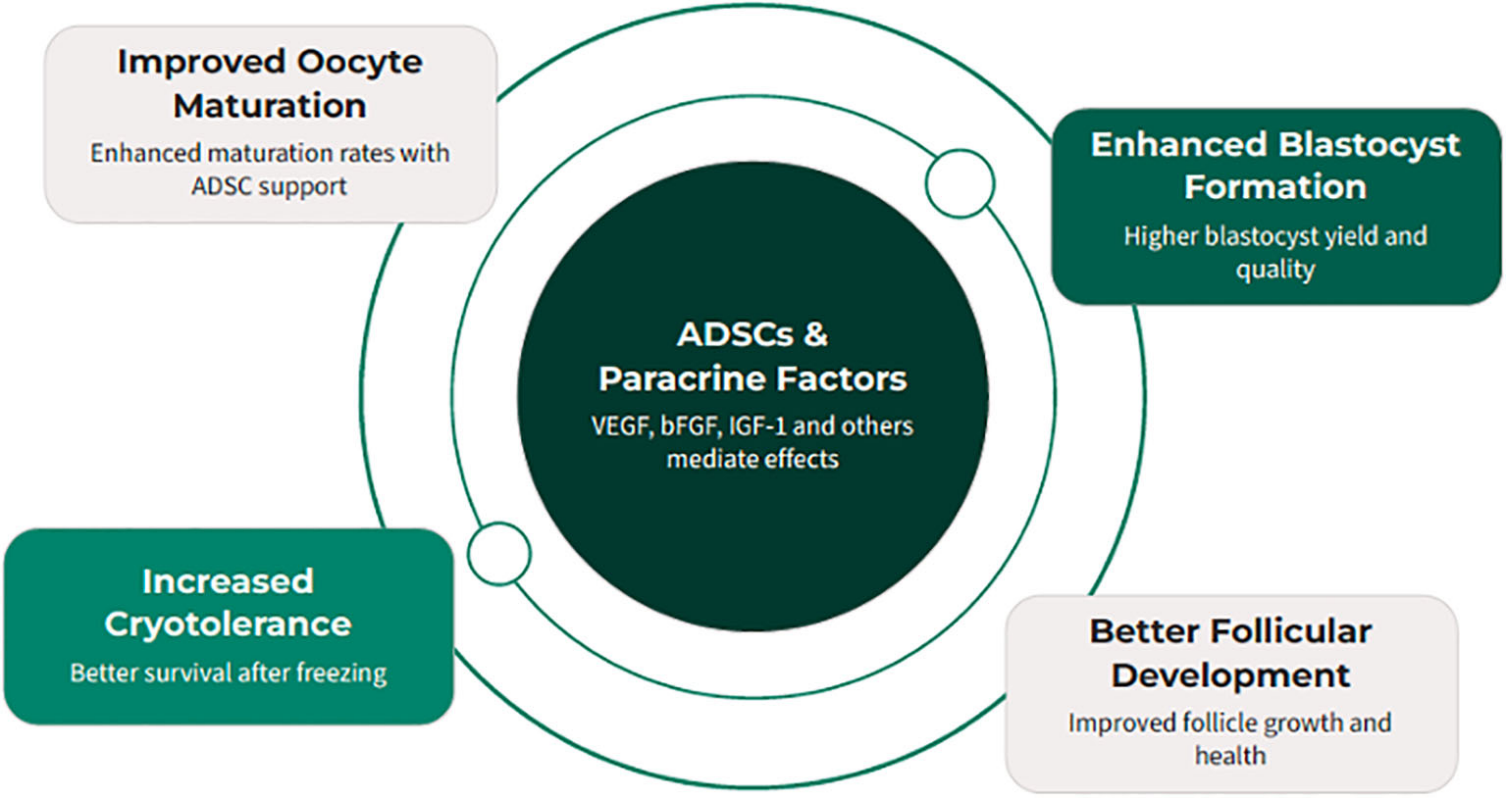
Adipose-derived stem cells (ADSCs) provide a paracrine-rich microenvironment that can significantly enhance various aspects of reproductive technology, from oocyte maturation to embryo development and follicular survival.

Early-stage follicles depend on paracrine signaling for survival and rarely thrive in isolation. ADSCs, which secrete growth and survival factors, were tested for their ability to support follicular development. In one study (44), primary and early secondary follicles from 10–12-day-old mice were co-encapsulated with ADSCs in an alginate 3D hydrogel for 14 days. Outcomes included survival, growth, antrum formation, meiotic competence, projections, and hormone production. ADSCs remained viable and significantly improved follicle survival (42.4–86.2% *vs*. <22% controls), growth, antrum formation, transzonal projections, and secretion of androstenedione, estradiol, and progesterone. Oocytes matured *in vitro* reached meiotic competence, with >90% undergoing GV breakdown and ~50% polar body extrusion (**Figure 4**). These findings indicate ADSCs create a paracrine-rich microenvironment that sustains follicular integrity, growth, and oocyte maturation. Limitations include reliance on a murine model, synthetic alginate scaffold, and lack of human validation. Future work should identify key ADSC-secreted factors and optimize biomaterials for fertility preservation.

ADSC-derived factors also influence gametes and embryos. Several models demonstrate that conditioned media, lysates, and EVs enhance oocyte maturation, embryo development, and cryotolerance. For example, ADSC lysates improved bovine blastocyst formation and survival after freezing, while exosome supplementation reduced oxidative stress and apoptosis in porcine embryos. Although porcine parthenogenetic studies have limited clinical relevance, they provide mechanistic insight into how ADSC-secreted factors modulate early development. Rodent embryo studies remain the most representative models for human reproductive biology and underscore the potential of ADSC-derived products to optimize embryo quality. Collectively, these findings suggest that ADSCs and their derivatives may improve multiple stages of reproduction—from follicle growth to embryo viability—through mechanisms that include paracrine signaling, mitochondrial transfer, and extracellular vesicle-mediated communication. **Table 2** summarizes available studies of ADSC-based therapies on ovarian function and embryo development.

**Table 2:** Ovarian applications of ADSCs.

Section	Indication / Model	Study type	N / Scale	Intervention (ADSC modality / route)	Key outcomes	Limitations
Mashayekhi et al. (33)	POI (women <40)	Phase I, first-in-human (non-randomized)	n=9 (3 dose cohorts)	Autologous ADSCs, intraovarian (US/laparoscopy); doses 5, 10, 15×10^6	No adverse events; 4/9 resumed menses; 4/9 FSH <25 IU/L (some up to 12 mo); variable AMH/AFC/ovarian volume	Small sample; no control; non-randomized; heterogeneous response; limited mechanistic insight
Takehara et al. (15)	CTX-induced ovarian failure (rat/mouse)	Preclinical animal (comparative controls: BMSC, fibroblasts)	Rodent models; F1/F2 follow-up	ADSCs intraovarian 15d post-CTX	↑ CD34+ angiogenesis; restored follicles & corpora lutea; litter size 13.6 *vs* 9.4; no malformations/tumors; ADSCs localized to theca (paracrine)	Rodent data; specific factors not fully defined
Wang et al. (17)	Poor oocyte quality due to aging (mouse)	Preclinical animal (oocyte-level experiments + embryo transfer)	GV/MII oocytes; ET: 51 *vs* 50 embryos	Microinjection of mitochondria isolated from autologous ADSCs into oocytes (with ICSI)	↑ mtDNA copy number; better spindle integrity; aneuploidy 4/12 *vs* 11/18; blastocysts 30% *vs* 15%; live pups 8/51 *vs* 1/50	Preclinical; long-term offspring safety unknown; scalability/ethics for humans uncertain
Jiang et al. (12)	Poor oocyte quality (human oocytes *in vitro*)	Preclinical human oocyte lab study (discarded immature oocytes)	Young (<35) & advanced-age (≥35) IVF patients' oocytes	Screened donor mitochondria (GC, ADSC, BMSC, USC); USC mitochondria co-injected at ICSI	USC mitochondria most oocyte-like; improved Δψm, ↓ ROS/Ca2+; ↑ embryo quality; higher euploidy in aged embryos (28.6% *vs* 0)	Small samples; no transfers/clinical outcomes; relies on discarded oocytes; long-term safety unknown
Bang et al. (39)	*In vitro* embryo development (porcine, parthenogenetic)	Preclinical animal (*in vitro* culture)	Parthenogenetic embryos	ADSC-derived EVs (1.5×10^9 particles/mL) added to culture	↑ blastocysts (28.6% *vs* 24.8%); ↑ GSH, ↓ ROS; ↑ mitochondrial activity; ↓ apoptosis; ↑ OCT4/NANOG/SALL4; proteomics: SRC–AKT–CDK1 implicated	Porcine parthenogenesis; no *in vivo* implantation or cross-species validation
Ren et al. (13)	CTX-induced POI (mouse)	Preclinical animal + *in vitro* (KGN cells)	Murine model; KGN cell assays	ADSC-derived exosomes (intraovarian); pathway interrogation with rapamycin	Restored ovarian histology, ↑ follicles & E2, ↓ FSH; ↓ ROS/MDA, ↑ SOD; ↓ GC apoptosis; suppressed autophagy via ↓ p-AMPK / ↑ p-mTOR	Murine xenograft; mouse ADSCs; short follow-up; no fertility outcomes
Huang et al. (40)	CTX-induced POI (mouse) + hGCs ex vivo	Preclinical animal + human GC assays	Murine model (4 wks); hGCs from POI patients	Human ADSC-derived exosomes (intraovarian)	Follicles restored (primordial→antral); E2/AMH/FSH normalized; hGCs: ↑ proliferation (76% *vs* 8%), ↓ apoptosis (4% *vs* 74%); ↑ SMAD2/3/5; knockdown abrogated effects	Murine model; short-term; exosomal cargo not fully defined; no reproductive endpoints
Lee et al. (41)	IVM & embryo development (porcine)	Preclinical animal (*in vitro*)	COCs; parthenogenetic activation	Human ADSC co-culture (transwell) or ADSC-conditioned medium	↑ maturation (~85% *vs* 78%); ↑ cleavage (~85% *vs* 82%); trend ↑ blastocysts (27% *vs* 18%); ↑ blastocyst cell number; enriched VEGF, bFGF, IGF-1, IL-10, EGF; ↑ GDF9/BMP15	Porcine oocytes; parthenogenesis; no *in vivo* validation
Manabe et al. (42)	Embryo culture & cryotolerance (bovine)	Preclinical animal (*in vitro*, 4 experiments)	Bovine IVF embryos	Bovine ADSC lysate (up to 10%) *vs* fibroblast lysate *vs* 10% FCS	↑ blastocysts (43.0% *vs* 29.9%); ↓ lipid accumulation *vs* FCS; better post-thaw survival/hatching; maintained cell viability; stable key gene expression	Bovine model; bioactive factors undefined; no ET outcomes
Miranda et al. (43)	Embryo development (bovine)	Preclinical animal (*in vitro*, 2 experiments)	IVP zygotes	Direct co-culture with bovine ADSCs (10^4/mL) *vs* SOF alone / preconditioned SOF / GC co-culture	↑ blastocysts (45.9% *vs* 19.0–25.0%); ↑ cell numbers; ↑ POU5F1 & G6PDH; no ↑ HSP70 *vs* GCs	Bovine; no transfer/*in vivo* data; specific factors not isolated
Green et al. (44)	Early follicles *in vitro* (mouse)	Preclinical animal (3D hydrogel culture)	Primary/early secondary follicles (85–115 μm) for 14 days	Co-encapsulation with murine ADSCs in alginate hydrogel	↑ survival (42.4–86.2% *vs* <22%); ↑ growth & antrum formation; ↑ TZPs; steroidogenesis (A4, E2, P4); meiotic competence (GVBD >90%, PB1 ~50%)	Murine model; synthetic scaffold; no human validation

POI, Premature Ovarian Insufficiency; HRT, Hormone Replacement Therapy; FSH, Follicle-Stimulating Hormone; AMH, Anti-Müllerian Hormone; AFC, Antral Follicle Count; CTX, Cyclophosphamide; BMSC, Bone Marrow-Derived Mesenchymal Stem Cells; FISH, Fluorescence In Situ Hybridization; VEGF, Vascular Endothelial Growth Factor; IGF, Insulin-Like Growth Factor; HGF, Hepatocyte Growth Factor; StAR, Steroidogenic Acute Regulatory Protein; MRT, Mitochondrial Replacement Therapy; GV, Germinal Vesicle; MII, Metaphase II; mtDNA, Mitochondrial DNA; ICSI, Intracytoplasmic Sperm Injection; GC, Granulosa Cells; USC, Urine-Derived Stem Cells; ROS, Reactive Oxygen Species; Ca²^+^, Calcium Ion; EV, Extracellular Vesicle; TEM, Transmission Electron Microscopy; qPCR, Quantitative Polymerase Chain Reaction; SRC, Proto-oncogene tyrosine-protein kinase Src; CDK1, Cyclin-Dependent Kinase 1; KGN, Human Granulosa-like Tumor Cell Line; E2, Estradiol; MDA, Malondialdehyde; SOD, Superoxide Dismutase; AMPK, AMP-Activated Protein Kinasel mTOR, Mechanistic Target of Rapamycin; SMAD, Mothers Against Decapentaplegic homolog; FCS, Fetal Calf Serum; IVM, In Vitro Maturation; COC, Cumulus–Oocyte Complex; CM, Conditioned Medium; SOF, Synthetic Oviductal Fluid; TZP, Transzonal Projection; GVBD, Germinal Vesicle Breakdown; PB1, First Polar Body; A4, Androstenedione; P4, Progesterone.

## 4 Discussion

The cumulative evidence reviewed highlights the broad regenerative potential of ADSCs and their derivatives in reproductive medicine. Across uterine, ovarian, and embryonic applications, ADSCs consistently demonstrated the ability to modulate critical biological processes such as angiogenesis, fibrosis, apoptosis, and oxidative stress. Preclinical models support the concept that ADSCs can act through both direct differentiation and paracrine mechanisms, including secretion of growth factors, exosomes, and functional mitochondria. These findings are particularly significant given the limitations of current infertility treatments, which often address symptoms rather than reversing underlying pathology.

Uterine applications of ADSCs, especially in models of IUA and thin EMT, show clear improvements in endometrial thickness, vascularization, and receptivity markers, with encouraging fertility outcomes in some animal studies. Early human studies, though small, report meaningful increases in endometrial thickness and implantation rates following ADSC or SVF infusion, suggesting translational feasibility. However, variability in delivery methods (intrauterine *vs.* transmyometrial), cell sources (fresh *vs.* cryopreserved), and use of scaffolds complicates interpretation and underscores the need for protocol standardization.

Ovarian applications have been investigated in both POI and chemotherapy-induced ovarian failure. Animal models consistently demonstrate follicular recovery, improved angiogenesis, and restored endocrine function following ADSC transplantation. Human studies remain limited to small phase I trials, but initial results show menstrual recovery and partial normalization of gonadotropin levels in some patients. Importantly, ADSC-derived mitochondria and exosomes expand the therapeutic scope by addressing age-related oocyte decline and chemotherapy-induced granulosa cell injury. These approaches offer innovative, cell-free or organelle-specific therapies that may bypass concerns about engraftment and tumorigenesis. Nevertheless, questions remain regarding the long-term functionality of restored ovarian tissue, durability of hormonal recovery, and potential epigenetic risks to offspring.

Embryo-level interventions involving ADSC-derived conditioned media, lysates, exosomes, and mitochondrial supplementation reveal promising improvements in oocyte maturation, blastocyst development, cryotolerance, and gene expression associated with pluripotency and metabolism. These findings suggest that ADSC-secreted factors can recreate aspects of the *in vivo* ovarian and oviductal microenvironment *in vitro*, offering potential adjuncts to optimize IVF and IVM outcomes. However, the translation of such strategies into clinical embryology requires caution, as most studies have been conducted in bovine or porcine models, and human validation is still lacking.

Despite these encouraging findings, the field faces several key challenges. First, methodological heterogeneity—including differences in ADSC isolation, characterization, dosage, route of administration, and adjunctive treatments—makes it difficult to compare outcomes across studies. Second, most available data derive from small sample sizes and short follow-up periods, limiting confidence in long-term safety and efficacy. Third, mechanistic insights remain incomplete, particularly regarding how ADSC-derived products interact with immune, vascular, and stromal components of the reproductive microenvironment. Fourth, regulatory and ethical considerations must be addressed, especially in the context of mitochondrial transfer and exosome therapy, where long-term heritable effects are unknown.

**Figure 5:**
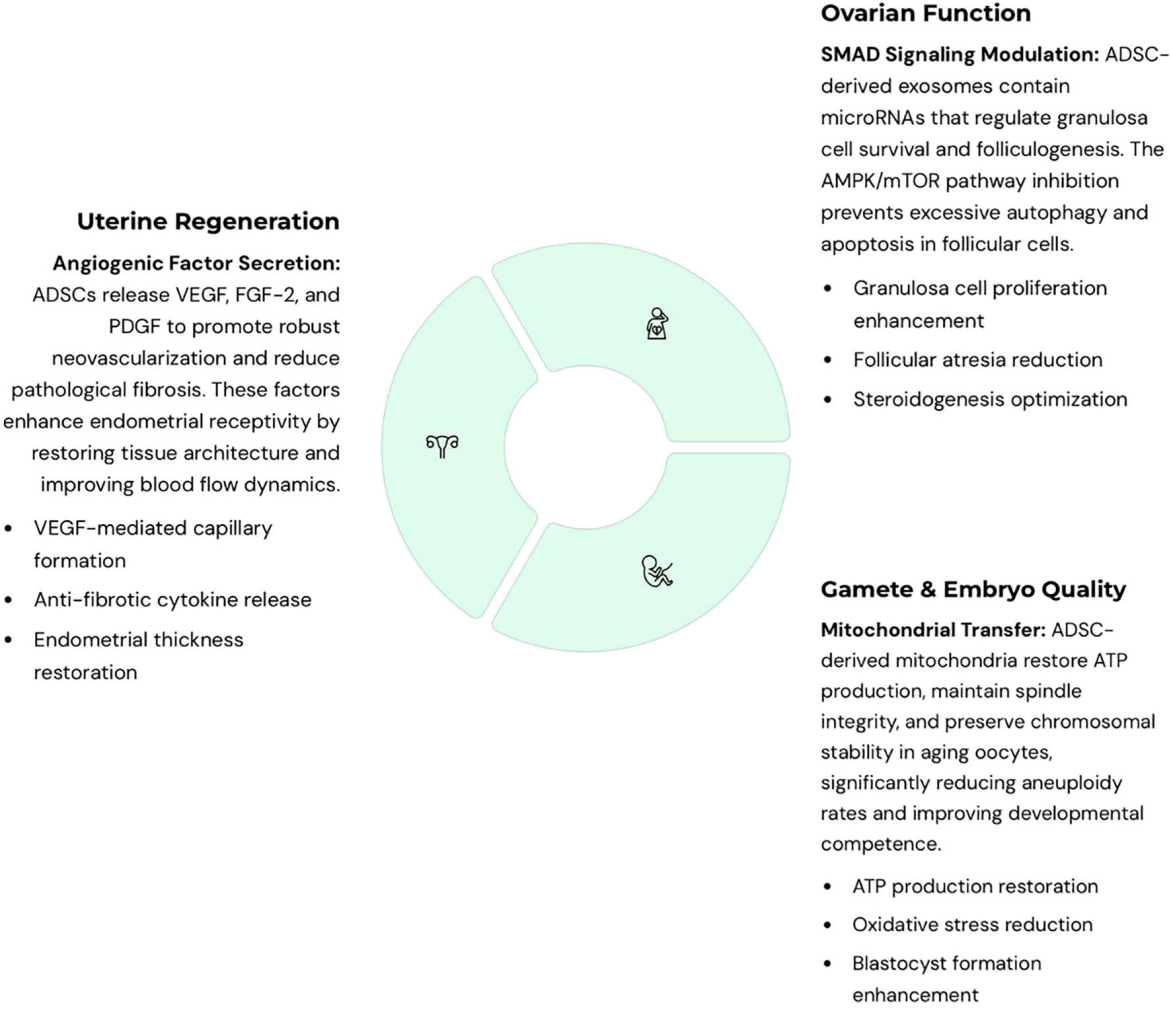
Integrated mechanistic pathways of adipose-derived stem cells (ADSC)-based therapies in reproductive medicine. ADSCs act through complementary mechanisms across uterine, ovarian, and embryonic applications. For the uterus, ADSCs secrete angiogenic factors such as VEGF that promote vascularization, reduce fibrosis, and enhance endometrial receptivity. For the ovaries, ADSC-derived exosomes regulate granulosa cell survival and folliculogenesis through SMAD signaling, while also inhibiting excessive autophagy and apoptosis via the AMPK/mTOR pathway. For oocyte quality and embryo development, ADSC-derived mitochondria restore ATP production, spindle integrity, and chromosomal stability in aging oocytes, reducing aneuploidy. Extracellular vesicles (EVs) and soluble cytokines further decrease oxidative stress, enhance blastocyst development, and improve cryotolerance. Collectively, these pathways converge to repair damaged reproductive tissues, improve gamete quality, and support early embryo development, highlighting the multifactorial regenerative potential of ADSCs.

Evidence suggests that ADSCs exert therapeutic effects through complementary mechanisms. First, paracrine signaling delivers growth factors, cytokines, and immunomodulatory molecules that reduce fibrosis, stimulate angiogenesis, and promote cell survival. Second, organelle transfer, particularly of mitochondria, restores bioenergetics and reduces meiotic errors in aging oocytes. Third, extracellular vesicles (exosomes) mediate intercellular communication by transferring proteins, nucleic acids, and metabolites that regulate apoptosis, oxidative stress, and autophagy. Together, these synergistic pathways highlight ADSCs’ unique ability to repair reproductive tissues at both cellular and molecular levels. Mechanistically, ADSCs modulate reproductive tissues through overlapping pathways: VEGF-driven angiogenesis in endometrium and ovary; SMAD signaling in granulosa cells promoting folliculogenesis; and AMPK/mTOR regulation of autophagy and apoptosis. Exosome cargo (microRNAs, growth factors) mediates many of these effects, while mitochondrial transfer restores oocyte energy balance. These mechanisms converge to reduce fibrosis, support follicular survival, and enhance embryo development (**Figure 5**).

Collectively, these limitations underscore the need for well-designed, multicenter randomized controlled trials with harmonized protocols, longer follow-up, and standardized outcome measures. Parallel mechanistic studies should dissect the molecular pathways activated by ADSCs, identify key paracrine mediators, and clarify their interactions with reproductive tissues. Only through such rigorous approaches can ADSC-based therapies move from experimental promise to clinical adoption.

Despite encouraging results, potential safety concerns must be carefully considered. Risks include immunogenic reactions to allogeneic preparations, and ectopic tissue formation at non-target sites. While no serious adverse events have been reported in early-phase studies, long-term surveillance in larger trials will be essential to establish safety before widespread clinical adoption.

## 5 Conclusion

ADSCs represent a versatile and promising platform for regenerative therapy in reproductive medicine. Evidence from preclinical studies and early human trials indicates that ADSCs and their derivatives can enhance endometrial receptivity, partially restore ovarian function, and improve oocyte and embryo quality. These benefits appear to be mediated through both cellular engraftment and paracrine mechanisms, including delivery of exosomes, cytokines, and mitochondria. Importantly, ADSCs are abundant, easily harvested, and generally well tolerated, making them attractive candidates for translational application.

However, the field remains in its early stages. The encouraging results observed to date must be validated in larger, controlled studies with standardized methodologies, mechanistic exploration, and long-term safety assessments, particularly regarding offspring health. If these challenges are addressed, ADSC-based therapies could become a transformative adjunct to assisted reproductive technologies, offering new hope to patients with refractory infertility due to endometrial injury, ovarian insufficiency, or poor oocyte quality. Ultimately, ADSCs may shift infertility treatment from symptomatic management toward true regenerative repair, aligning with the broader goals of personalized and precision reproductive medicine. Although preclinical findings are robust, clinical translation is still at an early stage, with only a few small pilot trials. Highlighting these limitations is essential for realistic appraisal of ADSC-based therapies.

## Data Availability

Publicly available datasets were analyzed in this study. This data can be found here: https://pubmed.ncbi.nlm.nih.gov.

## References

[1] Liang Y Huang J Zhao Q Mo H Su Z Feng S . Global, regional, and national prevalence and trends of infertility among individuals of reproductive age (15–49 years) from 1990 to 2021, with projections to 2040. Hum Reprod. (2025) 40:529–44. doi: 10.1093/humrep/deae292, PMID: 39752330

[2] Awonuga AO Camp OG Biernat MM Abu-Soud HM . Overview of infertility. Syst Biol Reprod Med. (2025) 71:116–42. doi: 10.1080/19396368.2025.2469582, PMID: 40117219

[3] Ethics Committee of the American Society for Reproductive Medicine . Child-rearing ability and the provision of fertility services: an Ethics Committee opinion. Fertil Steril. (2017) 108:944–7. doi: 10.1016/j.fertnstert.2017.10.006, PMID: 29202968

[4] Liu T He B Xu X . Repairing and regenerating injured endometrium methods. Reprod Sci. (2023) 30:1724–36. doi: 10.1007/s43032-022-01108-5, PMID: 36653588

[5] Maunder A Vermeulen N Vincent AJ Panay N Ee C . Complementary therapies for women with premature ovarian insufficiency: a systematic literature review to inform the 2024 update of the ESHRE/ASRM/IMS/CRE-WHiRL guidelines on premature ovarian insufficiency. Climacteric. (2025) 28:1–9. doi: 10.1080/13697137.2025.2530441, PMID: 40719542

[6] Martirosyan YO Silachev DN Nazarenko TA Birukova AM Vishnyakova PA Sukhikh GT . Stem-cell-derived extracellular vesicles: unlocking new possibilities for treating diminished ovarian reserve and premature ovarian insufficiency. Life (Basel). (2023) 13:2247. doi: 10.21873/invivo.11196, PMID: 38137848 PMC10744991

[7] Tsekouras A Mantas D Tsilimigras DI Moris D Kontos M Zografos GC . Comparison of the viability and yield of adipose-derived stem cells (ASCs) from different donor areas. In Vivo. (2017) 31:1229–34., PMID: 29102952 10.21873/invivo.11196 PMC5756658

[8] Zuk PA Zhu M Ashjian P De Ugarte DA Huang JI Mizuno H . Human adipose tissue is a source of multipotent stem cells. Mol Biol Cell. (2002) 13:4279–95. doi: 10.1091/mbc.e02-02-0105, PMID: 12475952 PMC138633

[9] Kim JH Choi SC Park CY Park JH Choi JH Joo HJ . Transplantation of immortalized CD34+ and CD34- adipose-derived stem cells improve cardiac function and mitigate systemic pro-inflammatory responses. PloS One. (2016) 11:e0147853. doi: 10.1371/journal.pone.0147853, PMID: 26840069 PMC4740491

[10] Zhao S Qi W Zheng J Tian Y Qi X Kong D . Exosomes derived from adipose mesenchymal stem cells restore functional endometrium in a rat model of intrauterine adhesions. Reprod Sci. (2020) 27:1266–75. doi: 10.1007/s43032-019-00112-6, PMID: 31933162

[11] Ai G Meng M Guo J Li C Zhu J Liu L . Adipose-derived stem cells promote the repair of chemotherapy-induced premature ovarian failure by inhibiting granulosa cells apoptosis and senescence. Stem Cell Res Ther. (2023) 14:75. doi: 10.1186/s13287-023-03297-5, PMID: 37038203 PMC10088140

[12] Jiang Z Shi C Han H Fu M Zhu H Han T . Autologous non-invasively derived stem cells mitochondria transfer shows therapeutic advantages in human embryo quality rescue. Biol Res. (2023) 56:60. doi: 10.1186/s40659-023-00470-1, PMID: 37978575 PMC10657142

[13] Ren Y He J Wang X Liang H Ma Y . Exosomes from adipose-derived stem cells alleviate premature ovarian failure via blockage of autophagy and AMPK/mTOR pathway. PeerJ. (2023) 11:e16517. doi: 10.7717/peerj.16517, PMID: 38107591 PMC10725676

[14] Sun M Wang S Li Y Yu L Gu F Wang C . Adipose-derived stem cells improved mouse ovary function after chemotherapy-induced ovary failure. Stem Cell Res Ther. (2013) 4:80. doi: 10.1186/scrt231, PMID: 23838374 PMC3854877

[15] Takehara Y Yabuuchi A Ezoe K Kuroda T Yamadera R Sano C . The restorative effects of adipose-derived mesenchymal stem cells on damaged ovarian function. Lab Invest. (2013) 93:181–93. doi: 10.1038/labinvest.2012.167, PMID: 23212100 PMC3561594

[16] Gharibeh N Aghebati-Maleki L Madani J Pourakbari R Yousefi M Ahmadian Heris J . Cell-based therapy in thin endometrium and Asherman syndrome. Stem Cell Res Ther. (2022) 13:33. doi: 10.1186/s13287-021-02698-8, PMID: 35090547 PMC8796444

[17] Wang ZB Hao JX Meng TG Guo L Dong MZ Fan LH . Transfer of autologous mitochondria from adipose tissue-derived stem cells rescues oocyte quality and infertility in aged mice. Aging (Albany NY). (2017) 9:2480–8. doi: 10.18632/aging.101332, PMID: 29283885 PMC5764387

[18] Shao X Ai G Wang L Qin J Li Y Jiang H . Adipose-derived stem cells transplantation improves endometrial injury repair. Zygote. (2019) 27:367–74. doi: 10.1017/S096719941900042X, PMID: 31452481

[19] Mouanness M Ali-Bynom S Jackman J Seckin S Merhi Z . Use of intra-uterine injection of platelet-rich plasma (PRP) for endometrial receptivity and thickness: a literature review of the mechanisms of action. Reprod Sci. (2021) 28:1659–70. doi: 10.1007/s43032-021-00579-2, PMID: 33886116

[20] Davar R Janati S Mohseni F Khabazkhoob M Asgari S . A comparison of the effects of transdermal estradiol and estradiol valerate on endometrial receptivity in frozen-thawed embryo transfer cycles: A randomized clinical trial. J Reprod infertility. (2016) 17:97–103. doi: 10.1097/MD.0000000000007720, PMID: 27141464 PMC4842240

[21] Wang L Huang X Li X Lv F He X Pan Y . Efficacy evaluation of low-dose aspirin in IVF/ICSI patients evidence from 13 RCTs: A systematic review and meta-analysis. Medicine. (2017) 96:e7720. doi: 10.1097/MD.0000000000007720, PMID: 28906358 PMC5604627

[22] Acharya S Yasmin E Balen AH . The use of a combination of pentoxifylline and tocopherol in women with a thin endometrium undergoing assisted conception therapies–a report of 20 cases. Hum fertility (Cambridge England). (2009) 12:198–203. doi: 10.3109/14647270903377178, PMID: 19938908

[23] Refai H Hassan D Abdelmonem R . Development and characterization of polymer-coated liposomes for vaginal delivery of sildenafil citrate. Drug delivery. (2017) 24:278–88. doi: 10.1080/10717544.2016.1247925, PMID: 28165805 PMC8241125

[24] Ke H Jiang J Xia M Tang R Qin Y Chen ZJ . The effect of tamoxifen on thin endometrium in patients undergoing frozen-thawed embryo transfer. Reprod Sci (Thousand Oaks Calif). (2018) 25:861–6. doi: 10.1177/1933719117698580, PMID: 28345485

[25] Zhao J Tian T Zhang Q Wang Y Li Y . Use of granulocyte colony-stimulating factor for the treatment of thin endometrium in experimental rats. PloS One. (2013) 8:e82375. doi: 10.1371/journal.pone.0082375, PMID: 24376532 PMC3871160

[26] Han X Ma Y Lu X Li W Xia E Li TC . Transplantation of human adipose stem cells using acellular human amniotic membrane improves angiogenesis in injured endometrial tissue in a rat intrauterine adhesion model. Cell Transplant. (2020) 29:963689720952055. doi: 10.1177/0963689720952055, PMID: 32838542 PMC7784510

[27] Dai Y Xin L Hu S Xu S Huang D Jin X . A construct of adipose-derived mesenchymal stem cells-laden collagen scaffold for fertility restoration by inhibiting fibrosis in a rat model of endometrial injury. Regener Biomater. (2023) 10:rbad080. doi: 10.1093/rb/rbad080, PMID: 37808957 PMC10551231

[28] Yotsumoto F Yoshikawa K Hirakawa T Urushiyama D Kiyoshima C Arima H . Safety and potential effect of intrauterine infusion of autologous adipose tissue-derived regenerative cells in patients with implantation failure: A pilot study. Cureus. (2024) 16:e57220. doi: 10.7759/cureus.57220, PMID: 38559528 PMC10980580

[29] Hernández-Melchor D Ortiz G Madrazo I Suarez JJ Barrera N Porchia LM . Improvement of endometrial thickness and *in vitro* fertilization outcomes in patients with Asherman's refractory endometrium using autologous mesenchymal stem cells from the stromal vascular fraction. Am J Transl Res. (2024) 16:4020–31. doi: 10.62347/UAGF1249, PMID: 39262711 PMC11384418

[30] Lee SY Shin JE Kwon H Choi DH Kim JH . Effect of autologous adipose-derived stromal vascular fraction transplantation on endometrial regeneration in patients of asherman's syndrome: a pilot study. Reprod Sci. (2020) 27:561–8. doi: 10.1007/s43032-019-00055-y, PMID: 32046396

[31] Panay N Anderson RA Bennie A Cedars M Davies M Ee C . Evidence-based guideline: premature ovarian insufficiency(†)(‡). Climacteric. (2024) 27:510–20. doi: 10.1080/13697137.2024.2408922, PMID: 39647506

[32] Ding C Zou Q Wang F Wu H Wang W Li H . HGF and BFGF secretion by human adipose-derived stem cells improves ovarian function during natural aging via activation of the SIRT1/FOXO1 signaling pathway. Cell Physiol Biochem. (2018) 45:1316–32. doi: 10.1159/000487559, PMID: 29462806

[33] Mashayekhi M Mirzadeh E Chekini Z Ahmadi F Eftekhari-Yazdi P Vesali S . Evaluation of safety, feasibility and efficacy of intra-ovarian transplantation of autologous adipose derived mesenchymal stromal cells in idiopathic premature ovarian failure patients: non-randomized clinical trial, phase I, first in human. J Ovarian Res. (2021) 14:5. doi: 10.1186/s13048-020-00743-3, PMID: 33407794 PMC7786909

[34] Chen H Liu C Zhu S Li S Zhang Q Song L . The therapeutic effect of stem cells on chemotherapy-induced premature ovarian failure. Curr Mol Med. (2021) 21:376–84. doi: 10.2174/1566524020666200905113907, PMID: 32888266 PMC8778633

[35] Cimadomo D Fabozzi G Vaiarelli A Ubaldi N Ubaldi FM Rienzi L . Impact of maternal age on oocyte and embryo competence. Front Endocrinol (Lausanne). (2018) 9:327. doi: 10.3389/fendo.2018.00327, PMID: 30008696 PMC6033961

[36] Fan XY Guo L Chen LN Yin S Wen J Li S . Reduction of mtDNA heteroplasmy in mitochondrial replacement therapy by inducing forced mitophagy. Nat BioMed Eng. (2022) 6:339–50. doi: 10.1038/s41551-022-00881-7, PMID: 35437313

[37] Alkhrait S Omran MM Ghasroldasht MM Park HS Katkhuda R Al-Hendy A . Exosome therapy: A novel approach for enhancing estrogen levels in perimenopause. Int J Mol Sci. (2024) 25:7075. doi: 10.3390/ijms25137075, PMID: 39000181 PMC11240923

[38] Mousaei Ghasroldasht M Liakath Ali F Park HS Hadizadeh M Weng SHS Huff A . A comparative analysis of naïve exosomes and enhanced exosomes with a focus on the treatment potential in ovarian disorders. J Pers Med. (2024) 14:482. doi: 10.3390/jpm14050482, PMID: 38793064 PMC11122298

[39] Bang S Qamar AY Yun SH Gu NY Kim H Han A . Embryotrophic effect of exogenous protein contained adipose-derived stem cell extracellular vesicles. J Anim Sci Biotechnol. (2024) 15:145. doi: 10.1186/s40104-024-01106-4, PMID: 39488683 PMC11531693

[40] Huang B Lu J Ding C Zou Q Wang W Li H . Exosomes derived from human adipose mesenchymal stem cells improve ovary function of premature ovarian insufficiency by targeting SMAD. Stem Cell Res Ther. (2018) 9:216. doi: 10.1186/s13287-018-0953-7, PMID: 30092819 PMC6085638

[41] Lee SH . Human adipose-derived stem cells' Paracrine factors in conditioned medium can enhance porcine oocyte maturation and subsequent embryo development. Int J Mol Sci. (2021) 22:579. doi: 10.3390/ijms22020579, PMID: 33430095 PMC7826973

[42] Manabe N Hoshino Y Himaki T Sakaguchi K Matsumoto S Yamamoto T . Lysate of bovine adipose-derived stem cells accelerates *in-vitro* development and increases cryotolerance through reduced content of lipid in the *in vitro* fertilized embryos. Biochem Biophys Res Commun. (2024) 735:150834. doi: 10.1016/j.bbrc.2024.150834, PMID: 39427378

[43] Miranda MS Nascimento HS Costa MP Costa NN Brito KN Lopes CT . Increasing of blastocyst rate and gene expression in co-culture of bovine embryos with adult adipose tissue-derived mesenchymal stem cells. J Assist Reprod Genet. (2016) 33:1395–403. doi: 10.1007/s10815-016-0779-0, PMID: 27475633 PMC5065556

[44] Green LJ Zhou H Padmanabhan V Shikanov A . Adipose-derived stem cells promote survival, growth, and maturation of early-stage murine follicles. Stem Cell Res Ther. (2019) 10:102. doi: 10.1186/s13287-019-1199-8, PMID: 30898159 PMC6427888

